# Potential and Actual Terrestrial Rabies Exposures in People and Domestic Animals, Upstate South Carolina, 1994–2004: A Surveillance Study

**DOI:** 10.1186/1471-2458-9-65

**Published:** 2009-02-23

**Authors:** Catherine W Roseveare, W David Goolsby, Ivo M Foppa

**Affiliations:** 1 Upstate Veterinary Specialists, 393 Woods Lake Road, Greenville, SC 29607, USA; 2 South Carolina Department of Health and Environmental Control, Region 2, 200 University Ridge, Greenville, SC 29602, USA; 3 Department of Epidemiology, School of Public Health and Tropical Medicine, Tulane University, 1440 Canal Street, New Orleans, LA 70112, USA

## Abstract

**Background:**

Although there has been a reduction of rabies in pets and domestic animals during recent decades in the United States, rabies remains enzootic among bats and several species of terrestrial wildlife. Spillover transmission of wildlife rabies to domestic animals therefore remains a public health threat

**Methods:**

Retrospective analysis of surveillance data of reported animal incidents (bites, scratches, mucous membrane contacts) from South Carolina, 1995 to 2003, was performed to assess risk factors of potential rabies exposures among human and animal victims.

**Results:**

Dogs and cats contributed the majority (66.7% and 26.4%, respectively) of all reported incidents, with stray dogs and cats contributing 9.0% and 15.1 respectively. Current rabies vaccination status of dogs and cats (40.2% and 13.8%, respectively) were below World Health Organization recommended levels. Owned cats were half as likely to be vaccinated for rabies as dogs (OR 0.53, 95% CI 0.48, 0.58). Animal victims were primarily exposed to wildlife (83.0%), of which 27.5% were rabid. Almost 90% of confirmed rabies exposures were due to wildlife. Skunks had the highest prevalence of rabies among species of exposure animals (63.2%). Among rabid domestic animals, stray cats were the most commonly reported (47.4%).

**Conclusion:**

While the majority of reported potential rabies exposures are associated with dog and cat incidents, most rabies exposures derive from rabid wildlife. Stray cats were most frequently rabid among domestic animals. Our results underscore the need for improvement of wildlife rabies control and the reduction of interactions of domestic animals, including cats, with wildlife.

## Background

Rabies in pets and domestic animals has been successfully reduced in the United States during recent decades with vaccination programs. Oral rabies vaccination (ORV) and other control programs in wildlife, on the other hand, have had limited success. Rabies remains enzootic among bats and several species of terrestrial wildlife [1,2], including raccoons (*Procyon lotor*), skunks (mostly *Mephitis mephitis*), and foxes (mostly *Vulpes vulpes*) [3]. Spillover transmission of wildlife rabies to domestic animals therefore remains a public health threat. Because there are few active surveillance efforts to find rabies in wildlife populations, the actual disease burden is likely underestimated in these populations [4].

There is currently no national surveillance for animal exposures, including bites, scratches, or mucous membrane contact with saliva or nervous tissue [5,6]. South Carolina, like some other states, mandates reporting of human victims, with voluntary reporting of animal victims. To characterize the epidemiology of potential rabies exposures in Upstate South Carolina, we conducted a retrospective study of reported animal incidents and compared potential and confirmed rabies exposures among human and animal victims.

## Methods

This study is based on Animal Incident/Rabies Investigation Reports collected by the South Carolina Department of Health and Environmental Control (SCDHEC) between 1994 and 2004 from Appalachia I, II, and III Public Health Districts that include the seven northern counties of Anderson, Cherokee, Greenville, Oconee, Pickens, Spartanburg, and Union. Such reports relate incidents of potential rabies exposures and include all reported animal bites, scratches, or potential mucous membrane contacts. Potential rabies exposures are reported by staff of medical treatment facilities, victims or bystanders (when animal victims are concerned) and are investigated by environmental health specialists with SCDHEC. Animal bites to humans are included in the South Carolina List of Reportable Conditions and is therefore mandated by law and regulation. Animal bites to animals are only reported voluntarily. As we were only concerned with terrestrial rabies, we excluded incidents involving bats (N = 245). If several people receive potential rabies exposures through the same animal a separate report results for each individual. Therefore, the number of Animal Incident/Rabies Investigation Reports may be different from the number of exposure animals.

Animal Incident/Rabies Investigation Reports were classified by victim type (human, animal species, or no victim). The "no victim" category was assigned to reported observations of atypical, erratic, or aggressive behavior of an animal, which occurred with no physical injury or mucous membrane exposure. Exposure animals were defined as animals implicated in an Animal Incident/Rabies Investigation Report. Dogs and cats were categorized according to ownership status (stray, pet). Vaccination status according to SCDHEC Animal Incident/Rabies Investigation Reports was verified by SCDHEC staff for each case and post-exposure prophylaxis (yes, no, unknown) was recorded. Stray animals, defined as those with no identified owner or individual harboring the animal, were considered unvaccinated. Similarly, dogs and cats with unknown immunization status were assumed to be unvaccinated. If a dog or a cat was classified as "stray", but was reported as having been vaccinated against rabies, it was excluded from analyses of ownership status.

All counties, with the exception of Cherokee county, utilize the dBase VIPER computerized database system. A database was created for Cherokee County data for 1998 – 2003 utilizing the original hard copy Animal Incident/Rabies Investigation Report forms. Use of computerized records varied by time period for some of the counties and resulted in 9 years of data from 3 counties, 5 years of data from two counties, and 3 years of data from two counties. Published SCDHEC laboratory confirmed animal rabies reports were utilized to confirm accuracy of county data. Animal rabies was diagnosed by direct fluorescent antibody testing of submitted brain tissues. Monoclonal antibody testing was used to determine the viral variant present in each positive sample.

Overall, 26 records were excluded from the analysis, because of inconsistencies, such as the specification of an animal species for the victim if the victim was human (N = 12) or no victim was reported (N = 4) or when no victim species was given for animal victims (N = 10). For rate calculations, 2000 census data was used.

To quantify bivariate associations between dichotomous exposure and outcome variables, we calculated odds ratios with asymptotic 95%-confidence intervals (CI) and associated p-values. For 22 contingency tables with small cell sizes we calculated exact confidence intervals for the odds ratios. For proportions, we calculated exact binomial confidence intervals. Proportions were compared using the Chi-square test for 2 × 2 contingency tables. We used an alpha level of 0.05. All statistical analyses were performed using SAS (Version 9, SAS Institute Inc., Cary, NC).

## Results

Overall, 22,485 animal incidence reports were included in this study. The vast majority of incidents involved human victims and accounted for 95.4% of the reports. The average annual incidence rate of incidents involving people was 203.7 per 100,000.

Dogs and cats most commonly caused incidents involving people (66.7% and 26.4%, respectively) (Table 1). Wild animals, such as raccoons, foxes, and skunks, on the other hand, accounted for only 1.7% of all human victims. Animal victims, predominantly dogs, were reported in 2.7% of all incidents, and no victim was associated with 2.0% of all reports. The majority (85.9%) of reports involving animal victims implicated wild species, especially raccoons as exposure animals.

**Table 1:** Potential exposure to terrestrial rabies in Upstate South Carolina, 1994–2004, classified by type of victim and exposure animal

		**Victim**					
**Exposure animal**	**Human**	**Dog**	**Cat**	**Other**^**‡**^	**None**	**Total (%)**

**Dog**	14,816	32	1	3	92	14,944 (66.7)
**Cat**	5,795	15	12	1	87	5,910 (26.4)
**Raccoon**	184	281	21	3	105	594 (2.7)
**Fox**	78	85	6	4	84	257 (1.1)
**Skunk**	14	72	0	0	21	107 (0.5)
Livestock*	28	0	0	0	5	33 (0.1)
**Other**^†^	541	20	1	0	5	567 (2.5%)

**Total**	21,456	505	41	11	399	22,412 (100.0%)

* Includes horses (N = 32) and one cow.

^† ^Includes ferrets (N = 11), rodents (N = 470) and rabbits (N = 86).

^‡ ^Includes cows (N = 4), horses (N = 4) and goats (N = 3); one incident in which a horse and a human were simultaneously indicated as victims, as well as eight incidents where the animal victim was not specified were excluded from this analysis.

**Figure 1:**
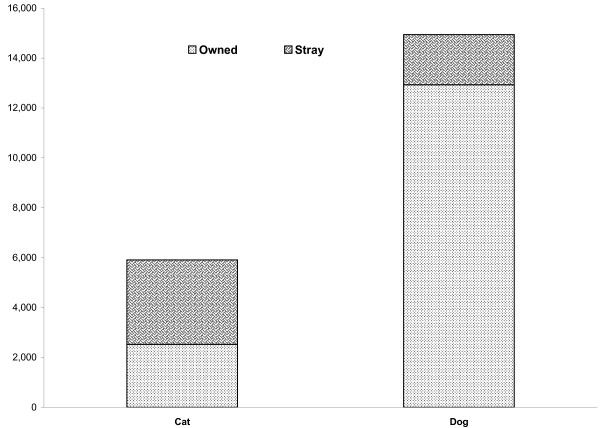
**Distribution of ownership status (owned vs. stray) among cats and dogs implicated as the exposure animal in incident reports, Upstate South Carolina, 1994–2004**.

Although dogs were overall more commonly implicated in incident reports than were cats, stray cats (N = 3,393) were more frequently reported than stray dogs (N = 2,017) (odds ratio 8.64, 95% C.I. 8.06, 9.26) (Figure 1).

Among owned dogs that were reported as exposure animals, rabies vaccination was current in almost half (46.5%), while in owned cats, that proportion was less than a third (32.3%) (odds ratio 1.66, 95% C.I. 1.47, 1.89) (Table 2). Rabid cats were much more likely to be strays than rabid dogs (odd ratio 14.4, 95% C.I. 1.5, 673.8) (Table 2)

**Table 2:** Vaccination status among dogs and cats reported as exposure animals in animal incident reports, Upstate South Carolina, 1994–2004.

	**Dogs**		**Cats**	
**Ownership**	**N (% Vaccinated)**	**Confirmed Rabid**	**N (% Vaccinated)**	**Confirmed Rabid**

**Pet**	12,927 (46.5)	10	2,517 (32.3)	9

**Stray**	2,012 (-^†^)	1	3,391 (-)	13

**All**	14,939	11	5,908	22

^† ^Stray animals were assumed to be unvaccinated.

Among all animals with a conclusive rabies test, 304, or one and a quarter of a percent, tested positive. Almost 90 percent of the rabid animals involved in incident reports were wildlife. Almost two thirds of the skunks with conclusive tests, and almost a third of the tested foxes and raccoons, were diagnosed with rabies (Table 3).

**Table 3:** Rabies prevalence among terrestrial animals with conclusive rabies results, Upstate South Carolina, 1994–2004.

	**N (pos.)**	**Prevalence %**^**‡**^ **(exact 95% confidence interval)**
**Raccoon**	472 (141)	29.9 (25.8, 34.2)
**Fox**	217 (68)	31.3 (25.2, 38.0)
**Skunk**	95 (60)	63.2 (52.6, 72.8)
**Domestic cat**	1826 (22)	1.2 (0.8, 1.8)
**Domestic dog**	1253 (11)	0.9 (0.4, 1.4)
**Livestock**^†^	14 (2)	14.3 (1.8, 42.8)
**Ferret**	2 (0)	0 (0, 84.2)
**Rodent**	196 (0)	0 (0, 1.9)
**Rabbit**	24 (0)	0 (0, 14.3)
**Total**	4099 (304)	7.4 (6.6, 8.3)

^† ^Includes 13 horses (two positive) and one cow.

^‡ ^Prevalence among those tested.

Animals were far more commonly exposed to rabid animals than people (odds ratio 112.8, 95% CI 83.7, 151.9). For animals, the most common source of rabies exposure was raccoons, followed by skunks, foxes and cat (Table 4). For people, on the other hand, the most common source of rabies exposure were foxes, followed by domestic cats, raccoons and dogs, skunks and livestock. All terrestrial animals were infected with the raccoon viral variant.

**Table 4:** Distribution of rabid exposure animals, by victim type, Upstate South Carolina, 1994–2004

		**Victim**					
		**Human**	**Dog**	**Cat**	**Other**^†^	**None**	**Total (%)**

**Exposure animal**	**Dog**	11	0	0	0	0	11 (3.6)
	**Cat**	19	0	1	1	1	22 (7.2)
	**Raccoon**	17	104	2	0	18	141 (46.4)
	**Fox**	34	23	0	1	10	68 (22.4)
	**Skunk**	8	45	0	0	7	60 (19.7)
	Livestock*	2	0	0	0	0	2 (0.7)
	**Other**	0	0	0	0	0	0 (0)

	**Total**	91	172	3	2	36	304 (100.0)

* Includes two horses.

^† ^Includes one cow and one horse.

## Discussion

Our results are consistent with some previous reports indicating that the majority of reported incidents in which an animal has potentially exposed a human to rabies are associated with pet dogs [7-10]. While cats were involved in fewer incidents overall than dogs, stray cats contributed to more incidents than stray dogs and were more frequently confirmed rabid. To our knowledge, a similar finding has not been previously reported. Most reported incidents to humans were from domestic animals, primarily dogs and cats, while the majority of reports concerning domestic animal victims involved wildlife (raccoons, skunks and foxes). Given a reported incident, animal victims were far more likely to be exposed to a rabid wild animal than human victims. Differences between animal and human victims are likely to be partially explained by strong reporting bias. Reporting mechanisms are inherently different for human and animal victims. While reporting of animal bites to humans is included in the South Carolina List of Reportable Conditions and thus mandated by law, incidents with animal victims are reported voluntarily [11]. Animal victims are often reported when rabies is specifically suspected in the exposure animal, which might bias estimates of rabies risk to animals associated with animal bites.

Overall, incidents in which an animal has potentially exposed another animal or human to rabies are likely to be under-reported [7]. Patrick et al. [9] estimated that up to half of all animal bites are not reported, with cat bites even less likely to be reported than dog bites. Animal victims may even be less likely to be reported as animal injuries are not officially reportable incidents and rabies is considered rare in the United States. This study found dogs much more frequently reported as victims of animal incidents than cats. It is currently unclear to what extent that difference reflects exposure risks of dogs and cats. While cats may not commonly be considered a potential source of rabies, outdoor domestic cats are often loosely owned and free roaming, potentially bringing them into contact with rabid wildlife [12,13]. In fact, a study of 31 rabid domestic cats in Maryland found that all were free-roaming [14]. Fight wounds in outdoor cats are a common injury [15], but unless a wild animal interaction is observed, the index of suspicion for rabies exposure is presumably low. Not surprisingly, therefore, rabid cats have outnumbered rabid domestic dogs in the United States since 1988 [3,12,16]. This finding was supported by our study.

The raccoon viral variant is the endemic terrestrial variant found in South Carolina and was the only rabies variant identified in this study. Raccoons were the most frequently tested exposure animals, which supports the finding by O'Bell and colleagues [17] for all of South Carolina. Foxes, on the other hand, were identified by our study as the most common source for rabies exposure to people, which is a new finding. Although dogs exposed people to rabies only rarely, dog bites trigger more than a third of all PEP courses [17]. Of all species, rabies tests in skunks had the highest *a priori *probability of being positive. This may be a result of case selection, as skunks are difficult to catch and unpleasant to handle. Skunks that are ill, possibly as a result of rabies, may be easier caught and thus submitted for testing. Skunks have been implicated as important hosts and vectors of raccoon rabies virus [12,18]. Further evaluation of the role of skunks in the transmission of raccoon rabies may provide vital information for the control of rabies in wildlife populations and the prevention of spillover transmission to domestic animals. Our findings are consistent with previous reports of rabid skunks frequently interacting with dogs, cats, and livestock [19] and underscore the importance of domestic animal injury surveillance for monitoring rabies in these populations.

The evidence of spill-over transmission of raccoon rabies to domestic animals in South Carolina is consistent with previous studies [20]. As a result of spillover transmission, domestic animals may become potential vectors for raccoon rabies to humans. Experimental studies indicate that cats are more susceptible than dogs to infection with rabies virus isolates from wildlife [15]. Our study indicates that rabid cats expose human victims to rabies about as frequently as rabid raccoons. Previous studies found that incidence of rabies in cats remained unchanged after the primary epizootic in raccoons subsided [12], which raises the question about the capacity of feral cats to act as rabies reservoir.

In our study, rabies vaccination coverage for both dogs and cats was lower than the 70% recommended by the World Health Organization (WHO) to effectively create herd immunity and prevent sustained transmission of rabies ([21,22]. Approximately, one half of owned dogs in our study were vaccinated in comparison to only one third of owned cats. Puppies and kittens less than 3 months of age are not yet vaccinated for rabies. If puppies and kittens were disproportionately included in our study, estimated vaccination coverage might be artificially deflated. Our estimates of vaccine coverage among cats are similar to the ones presented by Moore et. al [23]. Overall, the low rabies vaccination coverage in domestic animals is unlikely to be fully explained by bias.

The historical success of canine rabies vaccination in the US has created the public perception that the current risk of rabies is very low. However, the low vaccination coverage we found in domestic animals, and particularly cats, is cause for renewed efforts. Vaccination against rabies will help protect these animals and any mammalian contacts (people and other mammals). Lack of vaccination, on the other hand, will provide an opportunity for spillover transmission to domestic animals and their human companions. Veterinary rabies immunization protects and benefits both animal and human communities. The simplicity and proven efficacy of vaccination are the foundation of rabies prevention [21]. The increasing popularity of pet cats combined with the potential of cats to serve as vectors for wildlife rabies, substantiates the need for control strategies that specifically target free roaming and feral cats.

## Conclusion

While the majority of reported potential rabies exposures are associated with dog and cat incidents, most rabies exposures derive from rabid wildlife. Given a reported incident, domestic animal victims are much more likely to be exposed to rabies than people. Stray cats were disproportionately associated with human potential rabies exposures and were most frequently reported rabid among domestic exposure animals. Historically, rabies control has focused on vaccination and control of stray dogs. Our results underscore the need for future research to improve wildlife rabies control methods as well as the need for initiatives to reduce interactions of domestic animals, particularly cats, with wildlife.

## Competing interests

The authors declare that they have no competing interests.

## Authors' contributions

CR compiled the data, initiated the data analysis and manuscript preparation and wrote the first version of the manuscript; DG supervised the data compilation and helped write and edit the manuscript; IF supervised/conducted the analysis of the data and helped write and edit the manuscript and revised the manuscript according to reviewers' suggestions.

## Pre-publication history

The pre-publication history for this paper can be accessed here:


